# ANKRD26 and Its Interacting Partners TRIO, GPS2, HMMR and DIPA Regulate Adipogenesis in 3T3-L1 Cells

**DOI:** 10.1371/journal.pone.0038130

**Published:** 2012-05-30

**Authors:** Xiu-Fen Liu, Tapan K. Bera, Charissa Kahue, Thelma Escobar, Zhaoliang Fei, Gregory A. Raciti, Ira Pastan

**Affiliations:** Laboratory of Molecular Biology, Center for Cancer Research, National Cancer Institute, National Institutes of Health, Bethesda, Maryland, United States of America; University of Texas Health Science Center at Houston, United States of America

## Abstract

Partial inactivation of the *Ankyrin repeat domain 26* (*Ankrd26*) gene causes obesity and diabetes in mice and increases spontaneous and induced adipogenesis in mouse embryonic fibroblasts. However, it is not yet known how the Ankrd26 protein carries out its biological functions. We identified by yeast two-hybrid and immunoprecipitation assays the triple functional domain protein (TRIO), the G protein pathway suppressor 2 (GPS2), the delta-interacting protein A (DIPA) and the hyaluronan-mediated motility receptor (HMMR) as ANKRD26 interacting partners. Adipogenesis of 3T3-L1 cells was increased by selective down-regulation of *Ankrd26*, *Trio*, *Gps2, Hmmr* and *Dipa*. Furthermore, GPS2 and DIPA, which are normally located in the nucleus, were translocated to the cytoplasm, when the C-terminus of ANKRD26 was introduced into these cells. These findings provide biochemical evidence that ANKRD26, TRIO, GPS2 and HMMR are novel and important regulators of adipogenisis and identify new targets for the modulation of adipogenesis.

## Introduction

The current epidemic of obesity and diabetes has stimulated research to improve the understanding of these diseases and to identify and characterize new genes involved in their pathogenesis [1]–[5]. We recently demonstrated that the *Ankyrin repeat domain 26* (*Ankrd26*) gene plays an important role in the development of these pathologies. Mice with partial inactivation of the *Ankrd26* gene develop marked hyperphagia, severe obesity and an unusual form of diabetes in which white adipose tissue preserves its sensitivity to insulin [6], [7]. Moreover, when young *Ankrd26* deficient mice were placed on a pair-feeding diet with normal mice, they maintained normal body weight, showed a better glucose tolerance, and increased insulin sensitivity in the white adipose tissue, indicating a dual role of the *Ankrd26* gene in the control of appetite and of adipose tissue insulin sensitivity [7]. In addition, mouse embryonic fibroblasts (MEFs) from these mice have a higher rate of spontaneous adipogenesis compared to normal MEFs and their differentiation to adipocytes is greatly increased when they are exposed to a mixture of adipogenic inducers [8], indicating a prominent role of the *Ankrd26* gene in fat cells.

The Ankrd26 protein is highly expressed in the hypothalamus and other regions of the brain, as well as in many tissues and organs, including white adipose tissue. The protein is located in the cytosol close to the inner aspect of the cell membrane in HeLa and 293T cells expressing ANKRD26-EGFP, and it contains both ankyrin repeats and spectrin helices, through which it is potentially able to interact with other proteins [6]. However, it is not yet known what these proteins are and how the Ankrd26 protein carries out its biological functions.

In the present work, we used yeast two-hybrid and immunoprecipitation assays to identify interacting partners for the Ankrd26 protein, and performed knock-down experiments to establish whether any of these interacting proteins were functionally relevant in adipogenesis and whether this process was carried out by their interaction with Ankrd26 protein.

## Results

### Identification of ANKRD26 protein interaction partners

**Figure 1:**
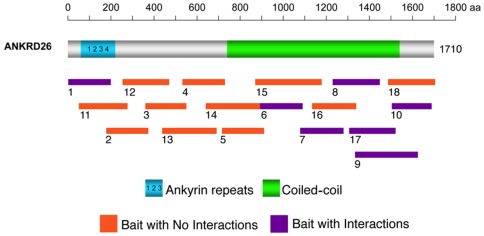
ANKRD26 yeast two-hybrid strategy. The position of ANKRD26's ankyrin and spectrin coiled-coil repeats are depicted on a schematic diagram of the full-length, 1710 amino acid, protein. The line below indicates the relative locations of ANKRD26 bait clones used in the *GAL4-*based yeast two-hybrid cDNA library screens. DIPA interacts with baits 7, 8, 9 and 17; GPS2 interacts with bait 17; HMMR interacts with baits 9 and 17; and TRIO interacts with bait 10.

To identify potential interacting partners of the ANKRD26 protein, we performed yeast two-hybrid screening using 18 baits, encoding overlapping fragments from the full-length ANKRD26 protein sequence (Figure 1). The baits were screened with three different cDNA expression libraries generated from human adult brain, human testis and mouse 11.5-day embryo, tissues known to express ANKRD26 [6]. Thirteen independent interacting proteins were identified by the screening, and 12 of them bound to the coiled-coil domain in the C-terminus, suggesting a prominent role of this domain for Ankrd26 protein-protein interactions (Table 1).

**Table 1:** Interactors for ANKRD26 identified by yeast two-hybrid screening.

cDNA Library	Identity	ANKRD26 Interacting Region (aa residues)	Description	Frequency†	Confirmed‡
**Human brain**	ANKRD17	1–199	Ankyrin repeat domain 17	1	ND§
**Human testis**	ANKRD18B	1320–1535	Ankyrin repeat domain 18B	3	ND
	C10RF9	1320–1535	Chromosome 1 open reading frame 9, isoform 1	1	ND
	GCC2	1344–1638	GRIP and coiled-coil domain containing 2, isoform a	1	ND
	LOC375010	1320–1535	Hypothetical protein	1	ND
**Mouse 11.5 days embryo**	Ccdc85b	1090–1638	Coiled-coil domain containing 85B	4	Yes
	Cxxc1	901–1100	CXXC finger 1	1	ND
	Fb1	1320–1535	Fb1	1	ND
	Gps2	1320–1535	G protein pathway suppressor 2	2	Yes
	HMMR	1320–1638	Hyaluron mediated motility receptor	2	Yes
	LOC66985	1344–1638	Rassf7 Ras association (RalGDS/AF-6) domain family 7	1	No
	Trio	1519–1702	Triple functional domain (PTPRF interacting), isoform 5	1	Yes
	Sdccag8	1090–1287	Serologically defined colon	1	ND

† Frequency: number of times pulled out during the two-hybrid screening.

‡ Confirmed: confirmation of screening results by co-immunoprecipitation.

§ ND: not done.

**Table 2:** Real Time Quantitative PCR primer sequences and primer efficiencies.

Primer Name	Orientation	Primer Sequences	Primer Efficiency
***Ankrd26***	Forward	5′-tgcccaggtaccatattcacga-3′	91
	Reverse	5′-tgcccaggtaccatattcacga-3′	
***Trio***	Forward	5′-gtgtgaccgcagtggataagagg-3′	91
	Reverse	5′-ccggggatactggcagggatgat-3′	
***Gps2***	Forward	5′-gggttgtggctgtggctgagatag-3′	95
	Reverse	5′-gggttgtggctgtggctgagatag-3′	
***Dipa***	Forward	5′-gctgctgcgggaaaatctgg-3′	86
	Reverse	5′-tccgggctgcccacactg-3′	
***Hmmr***	Forward	5′-gaagaaacaagctggggaggaa-3′	86
	Reverse	5′-aggggaagcaagccagtaagga-3′	
***Actin***	Forward	5′-acttgcggtgcacgatgga-3′	91
	Reverse	5′-tacccaggcattgctgacagg-3′	

### Validation of ANKRD26 protein interaction partners by IP

**Figure 2:**
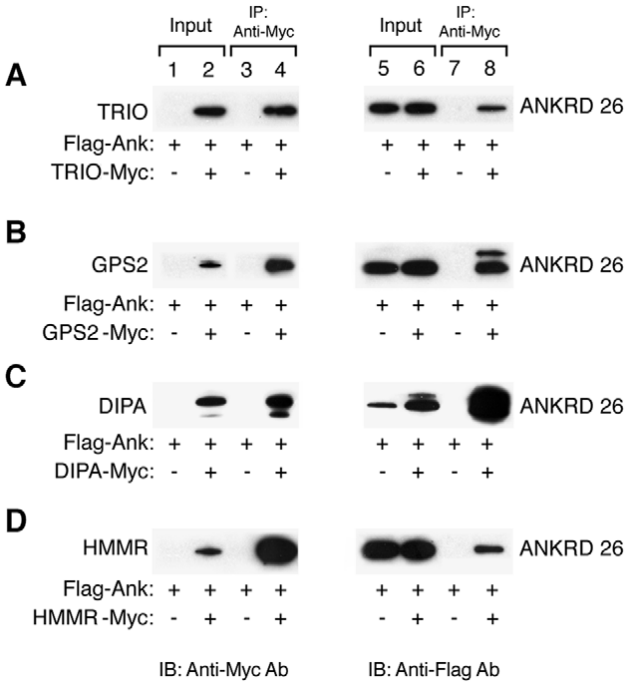
Protein interactions of ANKRD26 with TRIO, GPS2, DIPA and HMMR. **A**) Immunoblot of whole cell lysates (input; lanes 1–2 and 5–6) and IP (lanes 3–4 and 7–8) from 293/T cells over-expressing Flag-ANKRD26-C (lanes 1–8) and TRIO-Myc (lanes 2, 4, 6 and 8). **B**) Immunoblot of whole cell lysates (input; lanes 1–2 and 5–6) and IP (lanes 3–4 and 7–8) from 293/T cells over-expressing Flag-ANKRD26-C (lanes 1–8) and GPS2-Myc (lanes 2, 4, 6 and 8). **C**) Immunoblot of whole cell lysates (input; lanes 1–2 and 5–6) and IP (lanes 3–4 and 7–8) from 293/T cells over-expressing Flag-ANKRD26-C (lanes 1–8) and DIPA-Myc (lanes 2, 4, 6 and 8). **D**) Immunoblot of whole cell lysates (input; lanes 1–2 and 5–6) and IP (lanes 3–4 and 7–8) from 293/T cells over-expressing Flag-ANKRD26-C (lanes 1–8) and HMMR-Myc (lanes 2, 4, 6 and 8). IP were performed using the anti-Myc antibody as described in Methods. Left blots were probed with anti-Myc antibody, while right blots were striped and then probed with anti-Flag antibody.

Among the 13 interacting partners, we selected five to validate their interactions with ANKRD26 in mammalian cells. These are the triple functional domain protein (Trio), G-protein pathway suppressor 2 (Gps2), delta interacting protein A (DIPA; also called coiled-coil domain-containing protein 85B, Ccdc85b), hyaluronan-mediated motility receptor (HMMR), and Ras association (RalGDS/AF-6) domain family (N-terminal) member 7 (Rassf7; also referred to as LOC66985). Because all five partners interact with the C-terminus region of ANKRD26, we generated an expression plasmid containing the last 498 amino acids of human ANKRD26 (1212–1710 fragment) fused to a FLAG epitope tag located at the N-terminus of the protein (Flag-ANKRD26-C). Then a series of IP assays were performed in 293/T cells transiently co-transfected with Flag-ANKRD26-C and with different vectors, each expressing one of the selected proteins. IP analysis confirmed positive interactions for TRIO, GPS2, DIPA, and HMMR (Figure 2). No interaction was observed for Rassf7 (data not shown).

### ANKRD26/TRIO interaction by IP

Among the 13 interacting candidates, TRIO was pulled out once during the two-hybrid screening from the mouse embryo c-DNA library (Table 1). TRIO is a triple functional domain protein that promotes Ras homolog gene family (Rho) and Ras-related C3 botulinum toxin substrate 1 (Rac1) activation through the exchange of GDP by GTP [9]. Also with the protein tyrosine phosphatase (LAR) it plays a role in coordinating cell-matrix and cytoskeletal rearrangements necessary for cell migration and cell growth [10], [11]. To validate the TRIO interaction with ANKRD26 in 293T cells, we cloned the TRIO interacting region (aa 773–1059) into a pcDNA3.1 vector with a myc-epitope tag and co-transfected it with pFlag-ANKRD26-C into 293/T cells. Whole cell extracts were immunoprecipitated with anti-Myc antibody followed by immunoblot analysis with both anti-Flag and anti-Myc antibodies. Figure 2A shows that both TRIO-Myc (lane 2) and Flag-ANKRD26-C (lanes 5 and 6) proteins were expressed in 293T cells, and that the Flag-ANKRD26-C protein was efficiently immunoprecipitated by TRIO-Myc protein (lane 8), validating their interaction in mammalian cells.

### ANKRD26/GPS2 interaction By IP

GPS2 was pulled out two times from the mouse 11.5-day embryo cDNA library (Table 1). GPS2 is a protein involved in suppressing G-protein- and mitogen-activated protein kinase-mediated signal transduction [12], [13], and has been described as an integral subunit of the transcriptional repressor Nuclear CoRepressor-Hystone DeAcetylase 3 (N-CoR-HDAC3) complex [14]. To validate its interaction with ANKRD26 we co-transfected the Flag-ANKRD26-C and the Myc-tagged GPS2 vectors into 293/T cells and performed IP experiments, as described above. Figure 2B shows both proteins are detected in transfected 293/T cells (lanes 2, 5 and 6) and that the anti-Myc antibody efficiently pulled down Flag-ANKRD26-C protein as shown by the immunoblot with anti-Flag antibody (lane 8), validating the GPS2/ANKRD26-C interaction.

### ANKRD26/DIPA interaction by IP

DIPA was pulled out four times from a mouse 11.5-day embryo cDNA expression library (Table 1). DIPA, encoded by the CCDC85B gene, has a structural homology to hepatitis delta virus antigen (HDAg) [15]. DIPA has been reported as an inhibitory partner of the CCAAT-enhancer-binding protein (C/EBP)β and δ and has been implicated in adipocyte differentiation [16]. We co-transfected the Flag-ANKRD26-C expression vector with a Myc epitope tagged DIPA (DIPA-Myc) vector into 293/T cells. As shown in Figure 2C, DIPA-Myc and Flag-ANKRD26-C proteins are well expressed in 293T cell (lanes 2, 5 and 6), and the IP for the DIPA-Myc fragment specifically pulled down Flag-ANKRD26-C protein (lane 8). Similar results were obtained for IP experiment using an EGFP tagged ANKRD26-C construct (data not shown). Both data validate that a DIPA/ANKRD26-C interaction can occur in mammalian cells.

### ANKRD26/HMMR interaction by IP

During the two-hybrid screening HMMR was pulled out two times from the mouse 11.5-day embryo cDNA library (Table 1). The HMMR gene encodes a protein with a dual action. Within the cell it associates with microtubules and plays a role in the regulation of mitosis [17], while when released into the extracellular space, it can activate intracellular signaling pathways upon association with CD44 and hyaluronan [18]. Its interaction with ANKRD26 was validated by co-transfecting the Flag-ANKRD26-C vector and the Myc-tagged HMMR vector into 293/T cells. As shown in Figure 2D, both HMMR-Myc and Flag-ANKRD26-C proteins are detectable in transfected 293/T cells (lanes 2, 5 and 6) and the anti-Myc antibody efficiently pulled down Flag-ANKRD26-C (lane 8).

### Role of Ankrd26, Trio, Gps2, Hmmr and Dipa in adipogenesis

**Figure 3:**
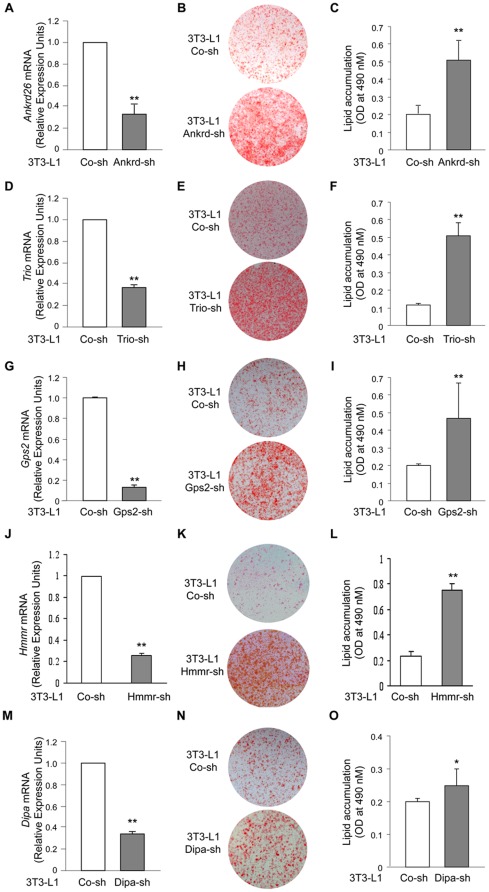
shRNA knockdown of *Ankrd26*, *Trio*, *Gps2, HMMR* and *Dipa* induces adipocyte differentiation in 3T3-L1 cells. To down-regulate *Ankrd26*, *Trio*, *Gps2, HMMR* and *Dipa* expression cells were transfected with specific shRNA for *Ankrd26* (Ankrd-sh), *Trio* (Trio-sh), *Gps2* (Gps2-sh), *HMMR* (HMMR-sh), *Dipa* (Dipa-sh) or with non-targeting control shRNA (Co-sh). **A**) *Ankrd26* mRNA expression determined by Real-Time Quantitative PCR analysis of total RNA isolated from 3T3-L1 cells transfected with Ankrd-sh or Co-sh. mRNA levels in Ankrd-sh treated cells are relative expression units to those in control (Co-sh; mean ± SD; n = 3). **p<0.01. Macroscopic images (**B**) and lipid quantification (**C**) in Ankrd-sh and Co-sh transfected cells stained with oil-red-O upon differentiation. Bars are expressed as means ± SEM of oil-red-O values measured at 490 nm. Ankrd-sh vs. Co-sh, **p<0.01. mRNA expression (**D, G, J** and **M**), macroscopic images (**E, H, K** and **N**) and lipid quantification (**F, I, L** and **O**) of 3T3-L1 cells transduced with Trio-sh (**D–F**), Gps2-sh (**G–I**), HMMR-sh (**J–L**) and Dipa-sh (**M–O**) were evaluated as **A**, **B** and **C**, respectively.

Because ablation of the C-terminus of Ankrd26 modulates adipogenesis in MEFs [8], we sought to establish whether Ankrd26 and/or any of its interacting partners have a role in differentiation of 3T3-L1 cells, which are widely used for adipogenesis differentiation studies [19]. The transduction of 3T3-L1 cells with a specific shRNA for *Ankrd26* (Ankrd-sh) resulted in a 65% reduction of *Ankrd26* mRNA compared to parental 3T3-L1 cells transfected with a non-specific shRNA (Co-sh) (Figure 3A). When differentiation was induced by an adipogenic cocktail for 5 days, *Ankrd26* down-regulation caused a 2.5-fold increase in adipogenesis compared to the control cells as shown by the accumulation of triglycerides in the cells (Figure 3B and C), indicating that the down-regulation of Ankrd26, as well as its partial ablation [8], modulates differentiation in fat cells.

We then focused our attention on ANKRD26 partners TRIO, which is a Rho and Rac activator and a LAR interacting partner [9]–[11], GPS2, and DIPA, which have been reported to control gene expression by inhibition of transcription [14], [16], [20] and HMMR, which can regulate cell migratory behavior and mitosis [17], [18]. In 3T3-L1 cells, *Trio* shRNA induced a 60% decrease of *Trio* mRNA levels (Figure 3D), and its down-regulation was paralleled by a 5-fold increase in adipogenesis compared with a control shRNA (Figure 3E and F). *Gps2* shRNA produced an 80% decrease of *Gps2* mRNA levels (Figure 3G), and this strong down-regulation was accompanied by a 3-fold increase in adipogenesis (Figure 3H and I). HMMR shRNA produced a 74% decrease in RNA levels (Figure 3J), which was accompanies by a 3-fold increase in adipogenisis (Figure 3K and L). Finally, when *Dipa* shRNA was transfected in 3T3-L1 cells, a 60% decrease of *Dipa* mRNA levels was observed (Figure 3M), and *Dipa* down-regulation caused a modest increase in adipogenesis (Figure 3N and O). These data confirm the role of DIPA as a negative regulator of adipogenesis [16], but more importantly they identify TRIO, GPS2 and HMMR as new proteins that also negatively regulate the adipogenic process in 3T3-L1 cells.

### GPS2 and DIPA localization in transfected 3T3-L1 cells

**Figure 4:**
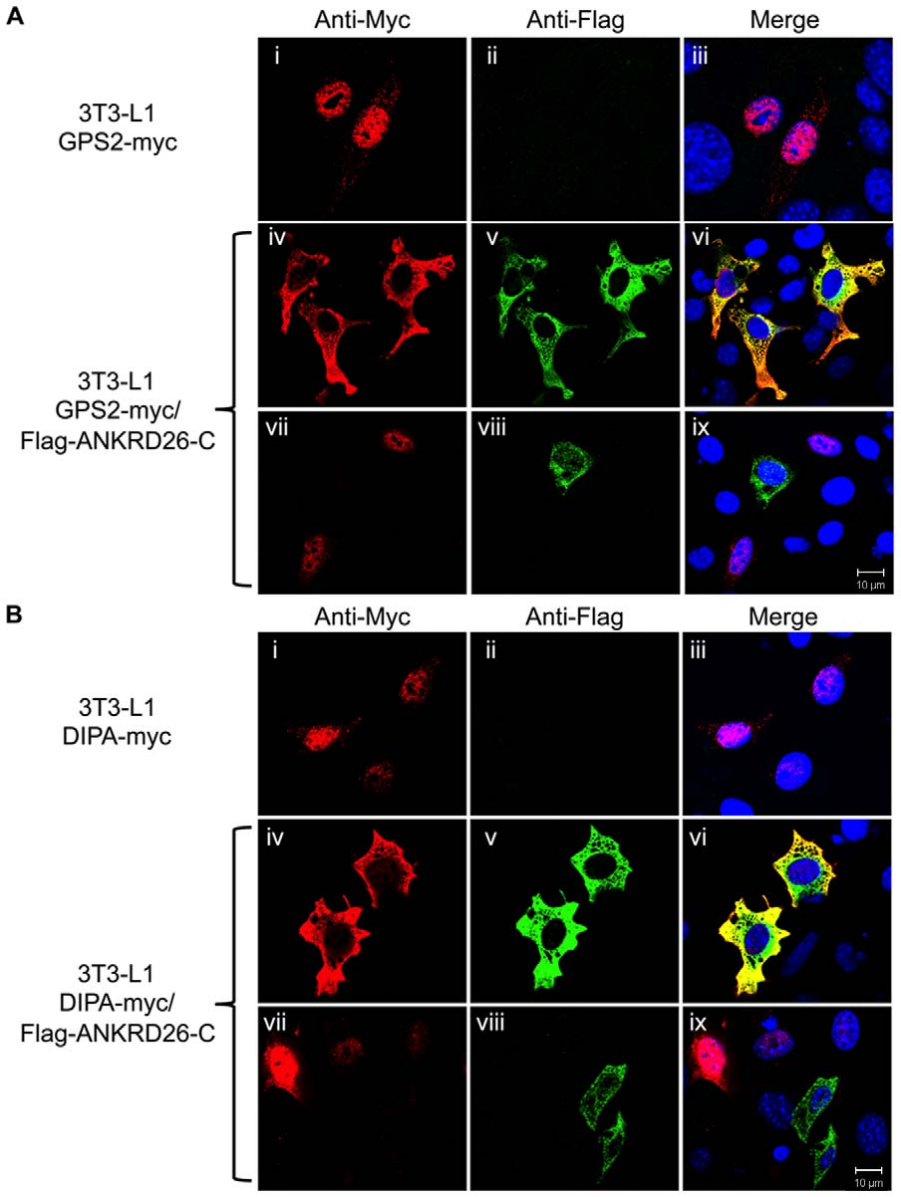
Nuclear localization of GPS2 and DIPA is affected in 3T3-L1 cells over-expressing Flag-ANKRD26-C. **A**) 3T3-L1 cells transfected with GPS2-Myc (i–ix) and Flag-ANKRD26-C vectors (iv–ix) were double stained with antibodies against anti-Myc (i, iv, vii) and anti-Flag (ii, v, viii) and with DAPI solution for the nucleus staining (iii, vi, ix). **B**) 3T3-L1 cells transfected with DIPA-myc (i–ix) and Flag-ANKRD26-C vectors (iv–ix) were stained as in **B**. The images labeled iii, vi and ix are the merged fluorescent images. Note that only co-transfected cells in Panels vii, viii and ix demonstrate cytosolic localization on GPS2 and DIPA.

Both GPS2 and DIPA have been previously found to localize to the nucleus [16], [20]. To investigate the effect of ANKRD26 on the localization of both GPS2 and DIPA proteins, confocal immunofluorescence studies were performed in 3T3-L1 cells individually over-expressing GPS2-Myc or DIPA-Myc proteins, or simultaneously over-expressing Flag-ANKRD26-C with GPS2-Myc or DIPA-Myc proteins. As shown in Figure 4Ai and Bi, both GPS2-myc and DIPA-myc proteins are localized in the nuclei of cells transfected with pcDNA3.1-GPS2-Myc and pcDNA3.1-DIPA-Myc, respectively. When co-transfected with Flag-ANKRD26-C, which is mainly located in the cytosol (Figure 4Av and viii), GPS2-Myc mostly co-localized with Flag-ANKRD26-C in the cytoplasm of 3T3-L1 cells (Figure 4Aiv–vi). Similarly, when the DIPA-Myc protein was expressed together with Flag-ANKRD26-C the location of DIPA was predominantly in the cytoplasm (Figure 4Biv–vi). These data confirm our finding that GPS2 and DIPA interact with ANKRD26 and provide a possible mechanism by which ANKRD26 can regulate the activity of these two proteins by removing them from the nucleus in 3T3-L1 cells.

We previously reported that full-length ANKRD26 is mainly located close to the plasma membrane, although some can be detected in the cytoplasm. To investigate if the full-length protein could decrease the amount of DIPA or GPS2 in the nucleus, cells were transfected with GPS2-myc or DIPA-myc and full-length ANKRD26. Unfortunately, very few transfected cells were found to express ANKRD26 and we could not get an answer to this question.

### Expression of Ankrd26, Trio, Gps2, HMMR and Dipa during adipogenic induction in 3T3-L1 cells

**Figure 5:**
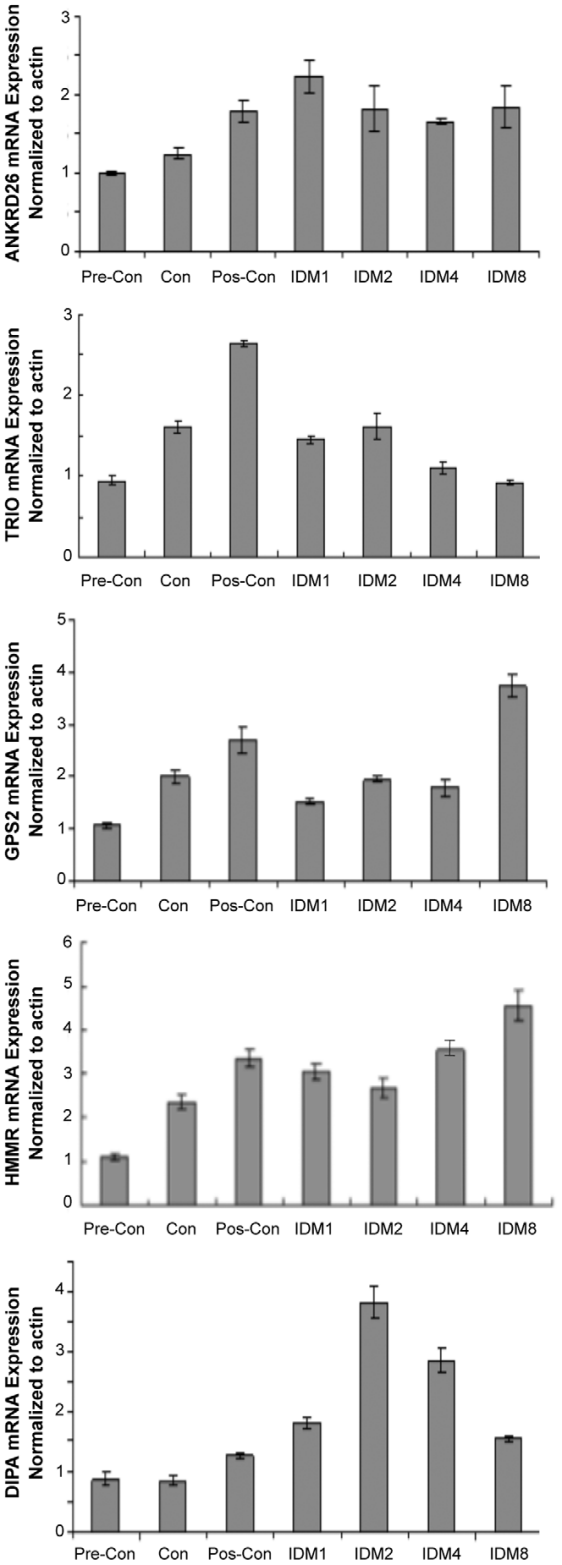
Analysis of *Ankrd26*, *Trio*, *Gps2, HMMR* and *Dipa* RNA by rq-pcr during adipogenesis induction. 3T3-L1 cells plated 6-well plates (1.8×10^5^/well) and harvested at various time points as: day 1 (pre-confluence), day 2 (confluence), day 4 (2 days after confluence), day 5 (IDM induction day 1), day 6 (IDM induction day 2), day 8 (IDM induction day 4), day12 (IDM induction day 8). RNA made by Trizol regent and subjected to Real Time Quantitative PCR using SYBR method. Bar represents triple for each sample.

To determine if the expression of endogenous Ankrd26 and its interacting partners were altered during adipogenesis in 3T3-L1 cells, we performed Real Time quantitative PCR using RNA isolated from 3T3-L1 treated with an adipogenic cocktail at various time points as described in the Methods. As shown in Figure 5, Ankrd26 expression increased 2-fold in the post-confluent cells as compared to pre-confluent condition and remains unchanged during adipogenic induction. Trio expression increased almost 3-fold in post-confluent condition and then remained at that level. Gps2 levels were increased at post-confluence but then fell to a pre-confluent level during early time points of cocktail treatment and then increased at day 8 as compared to pre-confluent cells. HMMR expression increased almost 3-fold in post-confluent condition and its levels remained high up to day 8. The expression of Dipa was increased almost 4-fold at day 2 of induction and remained high until day 4 but decreased on day 8 of adipogenic induction. These results indicate that the expression of *Ankrd26*, as well as the expression of its interacting partners is differentially regulated during adipogenesis.

## Discussion

In the present work, we identified 13 potential interacting partners for ANKRD26 by yeast two-hybrid screening, and validated as novel ANKRD26 interacting proteins TRIO, GPS2, DIPA, and HMMR by co-immunoprecipitation assays in mammalian cells. Furthermore, our analysis of adipogenesis showed that the selective down-regulation of *Ankrd26*, *Trio*, *Gps2, Hmmr* and *Dipa*, increased adipocyte differentiation upon induction in 3T3-L1 cells. These finding indicated that ANKRD26, TRIO, GPS2, HMMR and DIPA are regulators of adipogenesis. Further, GPS2 and DIPA proteins, that usually are located in the nucleus, become translocated into the cytoplasm when co-transfected with the C-terminus of ANKRD26 in 3T3-L1 cells, suggesting that the C-terminus may modulate the localization and function of these two proteins. However, due to our inability to transfect the very large full-length Ankrd26 cDNA into cells, we do not know if the full-length protein will have a similar interaction with the interacting proteins or change the nuclear localization of DIPA and GPS2. In addition we do not know if the interaction of one of the interacting partners affects the interaction of other interacting proteins with the carboxy-terminus of Ankrd26. Studies are underway to determine the binding site for each of the interacting proteins on Ankrd26.

The *ANKRD26* gene was originally identified as the ancestor of primate specific *ANKRD30A* and the *POTE* gene family [21]. It maps to chromosome 10p12.1 and encodes a protein of 192 kDa, which is located close to the inner aspect of the plasma membrane [6]. The ANKRD26 protein contains two conserved domains: Ankyrin repeats, located from amino acids 74–199, and helical regions (743–1538), which are structurally similar to the α-spectrin family proteins [6]. The presence of these two domains, known to mediate protein-protein interactions [22], [23], suggested that ANKRD26 might play a role as an adaptor protein [6]. From the two-hybrid screening data only one protein (ANKRD17) was found to interact with the ankyrin repeat region at the N-terminus of ANKRD26; all the other 12 proteins interacted with region 901–1702 of ANKRD26 that contains the spectrin-like coiled-coil domains. These findings indicate that the coiled-coil domain at the C-terminus of ANKRD26 rather than Ankyrin domain is mainly involved in ANKRD26 protein-protein interactions, and is likely to be responsible for its biological functions. In support of this last statement are our previous data obtained in mutant mice [6], [7], in which removal of the region encoding amino acids 1212–1710, encompassing part of the spectrin like helices and all the C-terminus of Ankrd26, cause extreme hyperphagia and severe obesity [6]. Furthermore, *Ankrd26* mutant mice develop an unusual form of obesity-induced diabetes in which white adipose tissue preserves its sensitivity to insulin in the context of whole body insulin resistance, and shows an improved sensitivity to the hormone when the mutant mice were pair fed with normal mice [7]. These data indicate that the C-terminus of Ankrd26 has at least two functions: one is to control the response to insulin in white adipose tissue; the other is to control appetite and satiety.

Recently we demonstrated that partial disruption of the *Ankrd26* gene regulates adipocyte differentiation both at the level of progenitor commitment and at early and late differentiation steps in MEF cells from *Ankrd26* mutant mice and that this process is associated with ERK-1/2 activation [8]. Here we show that down-regulation of *Ankrd26* expression caused an increase in adipogenesis when 3T3-L1 cells were transfected with a specific shRNA for the murine endogenous Ankrd26. All together these findings indicate that Ankrd26 has an important role in regulating differentiation of adipocytes.

The protein TRIO was initially identified as a binding partner for LAR, a trans-membrane protein tyrosine phosphatase. Its association with LAR was suggested to play a key role in the integration of diverse signals needed for cell-matrix interaction and cytoskeletal rearrangements during cell migration [9]–[11]. TRIO is a multidomain protein, which contains two guanine nucleotide exchange factor domains that promote Rho and Rac1 activation, and a serine/threonine kinase domain [9]. Furthermore, TRIO also possesses two pleckstrin homology domains that can recruit TRIO to different membranes, and four spectrin-like domains and an Ig-like domain that can mediate protein-protein interactions [9]. Here, we identified TRIO as a novel ANKRD26 interacting protein and we demonstrated for the first time the involvement of TRIO as negative regulator of adipogenesis in 3T3-L1 cells. Although the mechanism by which TRIO can inhibit adipogenesis, and how ANKRD26 can interfere with the function of TRIO are not yet known and are under investigation. We suggest that TRIO could regulate adipogenesis in combination with LAR and/or through the activation of Rho GTPase signaling. In support of this hypothesis are the recent reports that LAR and Rho signaling are negative regulators of adipogenesis [24], [25]. Kim *et al*. reported in human mesenchymal stem cells and in 3T3-L1 cells that the knock-down of endogenous LAR induced a dramatic increase of adipogenesis in these cells, while its over-expression caused a strong reduction of adipogenic differentiation by inhibition of insulin/insulin receptor signaling [24]. On the other hand, Sordella *et al*. reported that the modulation of Rho GTPase signaling regulates a switch between adipogenesis and myogenesis [25]. Indeed, mice lacking the Rho inhibitory protein p190-B RhoGAP exhibit a complete absence of mature adipocytes and MEF cells derived from these mice are defective in adipogenesis and this defect is rescued by inhibiting Rho kinase. Furthermore, the introduction of excessive Rho activity in MEF cells from control mice and in 3T3-L1 cells is sufficient to disrupt adipogenesis [25].

We have also shown that the selective down-regulation of the expression of three other ANKRD26 interacting partners, GPS2, HMMR and DIPA, enhances the proportion of differentiated cells. GPS2 was originally identified based on its ability to suppress lethal G protein subunit-activating mutations in the yeast pheromone responsive pathway [12], and its over-expression in mammalian cells potentially suppresses a RAS- and MAPK-mediated signal and interferes with JNK activity [12], [13]. Most importantly, GPS2 was found as an integral subunit of N-CoR-HDAC3 complex. It interacts directly with N-CoR and transducing beta-like protein 1 (TβL1) proteins inside the complex and it is essential for its assembly and stabilization [14]. It was recently demonstrated that the adipogenic transcription factor proliferator-activated receptor gamma (PPARγ) recruits N-CoR in the absence of its ligand, and that this co-repressor down-regulates PPARγ transcriptional activity. Furthermore, the silencing of N-CoR increases the expression of adipocyte specific genes and of adipogenesis in 3T3-L1 cells [26]. Similarly, PPARγ recruits HDAC3 to maintain transcriptional repression of its target genes [27]. Our findings demonstrate for the first time that GPS2 can act as a repressor of adipogenesis in 3T3-L1 cells and that its sequestration from the nucleus mediated by ANKRD26 may inhibit its function. Whether this regulatory process is mediated by GPS2 alone or in combination with the other proteins of the N-CoR-HDAC3 complex remain to be clarified.

The protein DIPA was initially identified as an interacting protein for the viral HDAg and it was shown to affect hepatitis D virus replication *in vitro* [15]. Bezy *et al*. recently described DIPA also as a novel interacting partner for the adipocyte transcription factors C/EBPβ and δ. They showed that over-expression of DIPA affects adipogenesis in 3T3-L1 cells; while its silencing enhances this process as we also showed in our study. Furthermore, they demonstrated that DIPA can co-localize with these two proteins in the nucleus of 3T3-L1 cells and may negatively modulate their transcriptional activity inhibiting adipogenesis [16]. Thus, our findings confirm the role of DIPA as negative regulator of this process and support the possible role of ANKRD26 as counter-regulator of DIPA mechanism by regulating DIPA localization.

HMMR is an oncogenic protein implicated in the progression of many human cancers, such as breast, gastric, and prostate cancers [28]. It was originally identified as a soluble protein, which binds to CD44 or hyaluronan and promotes cell motility and invasion through sustained stimulation of the activity of ERK1/2 kinase [18], [28]–[30]. Within the cell, HMMR plays a role in the regulation of mitosis through its association with microtubules [17], and it regulates the normal development of breast epithelial cells forming a complex with BRCA1 [31]. Furthermore it was recently shown that intracellular HMMR performs scaffolding functions to control both activity and targeting of ERK1/2 to tubulin [32]. Here, we identified HMMR as a novel ANKRD26 interacting partner and we showed for the first time its involvement as regulator of adipogenesis. Although how HMMR inhibits adipocyte differentiation and how ANKRD26 interferes with HMMR function remain to be clarified, we suggest that it could involve HMMR's ability to modulate ERK1/2 activation. It should be noted that the adipogenic induction in 3T3L1 is consistently higher during selective knock down of each of the interacting partners of Ankrd26 yet the endogenous expression of those proteins are not consistently lower during the adipogenic induction suggesting the involvement of multiple pathways and complex interaction of these interacting proteins in vivo.

In summary, we have identified several novel proteins that participate in the regulation of adipogenisis and we have shown that the region at the C-terminus of ANKRD26 that contains spectrin-like helices is responsible for its interaction with these proteins and in its regulation of adipogenesis. The identity of the residues that are responsible for the specific binding between ANKRD26 and its newly identified partners and the mechanism by which these proteins regulate adipogenisis are under investigation.

## Materials and Methods

### Yeast two-hybrid analysis

Yeast two-hybrid screening and analysis was performed by Myriad Genetics. Briefly, a bait construct expressing various fragments of ANKRD26 protein was fused to the C-terminus of a Gal4 DNA binding domain (residues 1–147). Each bait was transformed into the yeast strain PNY200. Prey constructs, expressing cDNA from human brain, testis or mouse embryo mRNA fused to the C-terminus of a Gal4 activation domain (amino acids (aa) 768–881), were transformed into yeast strain BK100. PNY200 cells (bait) were mated with BK100 cells (prey), and the desired diploid cells with positive interaction were selected with appropriate selectable marker. Interactions were further confirmed by transforming bait and prey constructs into naïve yeast cells and performing liquid culture β-galactosidase assays.

### 293/T cells: culture conditions and transient transfection

293/T cells (ATCC) were maintained in DMEM media (Quality Biologicals) supplemented with 10% FBS (Quality Biologicals), 2 mM L-Glutamine, 1 mM sodium pyruvate and 1% penicillin-streptomycin solutions in a humidified 5% CO_2_ incubator at 37°C. For transient transfection, expression vectors for pcDNA3.1-Flag-ANKRD26-C (aa 1212–1710), pcDNA3.1-DIPA-Myc, pcDNA3.1-GPS2-Myc, pcDNA3.1-TRIO-Myc and pcDNA3.1-HMMR-Myc were transfected individually or in combination into 293/T cells using lipofectamine reagent (Invitrogen) following the manufacturer's protocol. Cells were lysed 48 hr after transfection (see below).

### Cell lysate preparation, IP and western blot (WB) analysis

Both 293/T and 3T3-L1 (ATCC) cells were harvested and washed in PBS, and then lysed in Lysis Buffer containing 20 mM Tris-HCl, pH7.5, 150 mM NaCl, 5 mM EDTA, 1% NP40, 5 µg/ml leupeptin, 5 µg/ml aprotinin, 10 µM PMSF. Samples were incubated on ice 30 min after the addition of Lysis Buffer and then cell lysates were clarified by centrifugation at 15,000 g for 10 min at 4°C. The protein concentration of the cell lysate was determined using the Coomassie blue protein assay (Bio-Rad Laboratories). Protein lysates were then analyzed by SDS-PAGE, transferred to a PVDF membrane and subjected to WB analysis. For each IP experiment about 1 mg of protein lysate from 293/T cells transfected with the various combinations of vectors was incubated with 1.5 µg of anti-myc antibody (Sigma Aldrich) for 2 hr at 4°C. After incubation, 40 µl of protein A sepharose (Invitrogen) in a 1∶1 mixture of protein A sepharose and Lysis Buffer were added to the IP samples. Purified interacting complex was released from protein A sepharose beads by boiling in SDS-PAGE sample buffer and resolved on a 4–20% Tris-glycine gel. Samples were then subjected to WB analysis. Membranes were firstly probed with antibodies to Myc (Sigma Aldrich), Flag M2 (Sigma Aldrich), PPARγ (Santa Cruz Inc), C/EBPα (Cell Signaling), and β-tubulin (Sigma Aldrich) and then with secondary mouse or rabbit antibodies (GE Healthcare) before detection of the signal with ECL plus (GE Healthcare).

### Oil-red-o staining

Lipid droplets in differentiating or mature adipocytes were stained with the oil-red-O method as described [8]. Oil-red-O stained cells were scanned to obtain macroscopic pictures of cells. Cells were finally eluted with absolute isopropanol and the extracted red dye was measured with spectrophotometer at 490 nm.

### ShRNA and siRNA transduction

1.10^5^ 3T3-L1 cells were transduced with specific shRNA lentivirus particles for *Ankrd26* (TRCN0000254144), *Trio* (TRCN0000254107), *Gps2* (TRCN0000037101), *Dipa* (TRCN0000248678), HMMR (TRCN0000071589) and for the control shRNA SHC002V. All shRNA were purchased from Sigma Aldrich. After 3 days cells were selected with puromycin for 10 days. The puramycin-resistant cells were then collected, maintained in selection media and adipogenesis was induced as previously described.

### Immunofluorescence

3T3-L1 cells transfected with pcDNA3.1-Flag-ANKRD26-C, pcDNA3.1-DIPA-Myc and pcDNA3.1-GPS2-Myc expression vectors were fixed in 4% formalin solution, subjected to membrane permeabilization with PBS containing 0.1% TritonX-100, and then blocked for 1 hr in PBS containing 5% FBS and 3% BSA. Cells were than incubated with rabbit anti-myc and/or mouse anti-Flag antibodies for 2 hr at RT. After washing, cells were then incubated for 1 h with secondary antibody-fluorochrome-labeled (Alexa Fluor 594 anti-mouse IgG and Alexa Fluor 488 goat anti-rabbit IgG; Invitrogen). Cells were then washed and incubated in a DAPI solution for 10 min to stain the nucleus. Finally, slides were mounted with cover slip and observed on the Zeiss 510 confocal microscope (Carl Zeiss).

### Real time quantitative PCR

Total RNA extraction, cDNA synthesis and Real Time quantitative PCR were performed as described [8]. Quantitative PCR analysis of cells in the presence of corresponding shRNA was performed using mRNA isolated from cells before adding the adipogenic cocktail for differentiation. Primer sequences are listed in Table 2.

### Statistical analysis

Data are expressed as means ± SEM and statistical significance between groups was analyzed by 2-tailed Student's t test. *P* values of <0.05 were considered statistically significant.
